# Quantitative MRI Measures and Cognitive Function in People With Drug-Resistant Juvenile Myoclonic Epilepsy

**DOI:** 10.1212/WNL.0000000000209802

**Published:** 2024-09-20

**Authors:** Bernardo Crespo Pimentel, Giorgi Kuchukhidze, Fenglai Xiao, Lorenzo Caciagli, Julia Hoefler, Lucas Rainer, Martin Kronbichler, Christian Vollmar, John S. Duncan, Eugen Trinka, Matthias J. Koepp, Britta Wandschneider

**Affiliations:** From the Department of Neurology, Neurointensive Care and Neurorehabilitation (B.C.P., G.K., J.H., L.R., E.T.), Neuroscience Institute (B.C.P., G.K., J.H., M.K., E.T.), and Department of Child and Adolescent Psychiatry (L.R.), Christian Doppler University Hospital, Paracelsus Medical University, Centre for Neuroscience Salzburg, Member of the European Reference Network, EpiCARE, Austria; Department of Clinical & Experimental Epilepsy (B.C.P., F.X., L.C., J.S.D., M.J.K., B.W.), UCL Queen Square Institute of Neurology, London; Chalfont Centre for Epilepsy (B.C.P., F.X., L.C., J.S.D., M.J.K., B.W.), Chalfont St. Peter, United Kingdom; Department of Neurology (L.C.), Inselspital, Sleep-Wake-Epilepsy-Center, Bern University Hospital, University of Bern, Switzerland; Department of Psychology (M.K.), University of Salzburg, Austria; Department of Neurology (C.V.), Epilepsy Center, University Hospital of the Ludwig-Maximilians-University of Munich, Germany; Department of Public Health, Health Services Research and Health Technology Assessment (E.T.), UMIT-University of Health Sciences, Medical Informatics and Technology, Hall in Tirol; and Karl Landsteiner Institute for Neurorehabilitation and Space Neurology (E.T.), Salzburg, Austria.

## Abstract

**Background and Objectives:**

Neuroimaging studies have so far identified structural changes in individuals with juvenile myoclonic epilepsy (JME) when compared with controls. However, the underlying mechanisms of drug-resistant JME remain unknown. In this study, we aimed at characterizing the structural underpinnings of drug-resistant JME using MRI-derived cortical morphologic markers.

**Methods:**

In this prospective cross-sectional 2-center study, T1-weighted MRI and neuropsychological measures of verbal memory and executive function were obtained in individuals with drug-resistant and drug-responsive JME recruited from epilepsy outpatient clinics and healthy controls. We performed vertexwise measurements of cortical thickness, surface area, and local gyrification index (LGI). Vertexwise group comparisons were corrected for multiple comparisons at a familywise error (FWE) of 0.05. The neuropsychological profile of disease subgroups was analyzed through principal component analysis.

**Results:**

We studied 42 individuals with drug-resistant JME (mean age 29 ± 11 years, 50% female), 37 with drug-responsive JME (mean age 34 ± 10, years, 59% female), and 71 healthy controls (mean age 21 ± 9 years, 61% female). Surface area was increased in participants with drug-resistant JME in the left temporal lobe (Cohen *d* = 0.82 [−0.52 to −1.12], *p*_FWE_ < 0.05) when compared with the drug-responsive group. Although no cortical thickness changes were observed between disease subgroups, drug-resistant and drug-sensitive participants showed discrete cortical thinning against controls (Cohen *d* = −0.42 [−0.83 to −0.01], *p*_FWE_ < 0.05; Cohen *d* = −0.57 [−1.03 to −0.11], *p*_FWE_ < 0.05, respectively). LGI was increased in the left temporal and occipital lobes in drug-resistant participants (Cohen *d* = 0.60 [0.34–0.86], *p*_FWE_ < 0.05) when contrasting against drug-sensitive participants, but not controls. The composite executive function score was reduced in drug-resistant individuals compared with controls and drug-sensitive individuals (−1.74 [−2.58 to −0.90], *p* < 0.001 and −1.29 [−2.25 to −0.33], *p* < 0.01, respectively). Significant correlations were observed between executive function impairment and increased surface area in the precuneus and medial prefrontal regions (*r* = −0.79, *p*_FWE_ < 0.05) in participants with drug-resistant JME.

**Discussion:**

We identified a developmental phenotype in individuals with drug-resistant JME characterized by changes in cortical surface area and folding complexity, the extent of which correlates with executive dysfunction. No association was observed between cortical thickness and disease severity. Our findings support a neurodevelopmental basis for drug resistance and cognitive impairment in JME.

## Introduction

Juvenile myoclonic epilepsy (JME) is the most common idiopathic generalized epilepsy (IGE) syndrome in adults, affecting 5%–10% of all people with epilepsy.^1^ Good treatment response was long regarded as a core characteristic of IGE syndromes. This feature was dropped from the International League Against Epilepsy 2022 classification of epilepsy syndromes^2^ in an attempt to include drug-resistant individuals under the same umbrella.^3^ Similar to focal epilepsies, up to 30% of people with JME are refractory to antiseizure medications (ASMs).^4,5^ Individuals with drug-resistant IGE syndromes also tend to present with seizures at a younger age and have a higher incidence of psychiatric comorbidity and cognitive impairment, mainly affecting frontal lobe functions such as working memory and executive function.^5-8^ By definition, clinical imaging is unremarkable in IGE. Multiple large-scale MRI and genetic studies have not yet supported the concept of a distinct structural phenotype associated with drug resistance.^9-11^ Such studies have, however, mostly focused on individuals with IGE syndromes in whom seizures persist into adulthood and thus represent cohorts who are unlikely to achieve disease remission.

Quantitative MRI measures of cortical thickness and other brain surface metrics deliver robust and reproducible markers of neurodegeneration and neurodevelopment.^12,13^ Neuroimaging studies in people with JME mainly identified structural anomalies in thalamic, premotor, and medial prefrontal areas,^14-18^ but also more recently including the mesiotemporal lobe.^19,20^ It remains unclear whether such abnormalities represent disease developmental signatures or are rather consequences of the disease. The concept of endophenotype (i.e., disease trait that is more prevalent in patients and first-degree relatives than the general population) has been used in imaging studies to disentangle characteristics attributable to the genetic underpinnings of disease from the effects of disease activity or ASM. Notably, in a recent endophenotype study, we identified changes in surface area and sulcal-gyral complexity as neurodevelopmental markers in JME.^13^ Because cortical surface characteristics are believed to be highly heritable,^21^ we hypothesize that alterations of cortical architecture may reflect a continuum determined by different degrees of genetic vulnerability and manifesting in distinct clinical phenotypes, possibly explaining intractability in people with JME.

In this study, we aimed to identify structural cortical correlates of drug resistance in a large multicentric JME cohort. We used structural MRI to provide quantitative measures of cortical organization and folding that reflect brain development. Neuropsychological tests were implemented to address verbal memory and executive function and thus characterize the cognitive phenotype of participants with drug-resistant JME. We then assessed the relationship between domain-specific cognitive performance and changes in cortical surface features to identify developmental alterations that underpin both disease severity and cognitive impairment.

## Methods

### Participants

In this cross-sectional study, we included a total of 79 participants with JME recruited from epilepsy outpatient clinics at the Department of Neurology, Christian Doppler University Hospital in Salzburg, Austria (CDK) (51 patients, 59 controls), and University College London Hospitals, London, United Kingdom (UCL) (28 patients, 19 controls), between 2007 and 2019. People with JME were divided into drug-resistant (n = 42) and drug-responsive (n = 37) groups. Drug resistance was defined as ongoing seizures in the 12 months before MRI acquisition despite at least 2 appropriate and well-tolerated ASM trials.^22^ 71 healthy controls comparable for age and sex were considered for analysis. All participants had an otherwise unremarkable neurologic history. MRI scans were reported as normal by a neuroradiologist. Results on cortical thickness and surface area were previously reported for all 28 individuals with JME and 19 healthy controls scanned at UCL.^13^

Apart from clinical and demographic metrics, we quantified the total ASM load by calculating the ratio of the actual daily dose of a specific ASM by its defined daily dose, provided by the Collaborating Centre for Drug Statistics Methodology of the World Health Organization, as previously reported.^23^ The cumulative ASM load for each individual is the sum of this ratio for all ASMs of the individual drug regimen.

### Standard Protocol Approvals, Registrations, and Patient Consents

All study procedures were performed in accordance with the Declaration of Helsinki. The study was approved by the corresponding ethics committee of the UCL Queen Square Institute of Neurology and University College London Hospitals, as well as the region of Salzburg. All participants provided written informed consent.

### Data Acquisition and Preprocessing

Structural MRI data at the CDK were obtained with a 3T Magnetom Prisma-Fit 3T MRI scanner using a 3-dimensional (3D) multiecho magnetization prepared rapid gradient-echo imaging sequence (repetition time 2.4 milliseconds, echo time 2.2 milliseconds, inversion time 1,060 milliseconds, flip angle = 8°, matrix = 320 × 300, voxel size = 0.8 × 0.8 × 0.8 mm^3^). Participants from UCL were scanned using a GE MR750 3T MRI scanner with a 3D fast spoiled gradient-echo sequence (repetition time 7.2 milliseconds, echo time 2.8 milliseconds, inversion time 450 milliseconds, flip angle = 20°, matrix = 256 × 256, voxel size = 1.1 × 1.1 × 1.1 mm^3^). Both MRI data sets were processed with the standardized FreeSurfer processing pipeline.^24^ The processing stream consisted of automated transformation to Talairach space, skull stripping, intensity normalization, and segmentation of white/gray matter tissue, resulting in extraction of surface meshes composed of approximately 16,000 vertices in each hemisphere. Surface extractions were visually assessed and manually corrected. Individual surfaces were then registered to an average template surface.

### Computation of Surface Parameters

Building on previous studies on epilepsies^13,15,25^ and to consider the 3D properties of the human cortex, we focused our analysis on cortical surface markers, including cortical thickness, white matter surface area, and local gyrification index (LGI). Cortical volume was omitted from the analysis because it is a function of both surface area and cortical thickness.^26^ Cortical thickness was calculated as the distance between corresponding vertices in the white matter and pial surfaces. In line with previous work, the surface area was calculated based on the average area of 6 triangles surrounding the index vertex on the white matter surface, thus reflecting the vertexwise degree of cortical expansion or compression.^27^ LGI was computed at each vertex using an inbuilt FreeSurfer function that quantifies the gyrification index in a 3D framework.^28^ LGI represents the amount of cortex buried within the sulcal folds compared with the amount of visible cortex across the whole cortical surface in each vertex. Cortex with extensive folding has a large gyrification index, whereas a cortex with limited folding has a small gyrification index. Cortical thickness and surface area were smoothed with 10-mm, 15-mm, and 20-mm surface-based kernels, whereas a kernel of 5 mm was applied to LGI because of previous inherent smoothing in metric calculation. Changing the diffusion kernel for cortical thickness and surface area estimates did not alter our group comparison results. Therefore, and to comply with previous literature and preserve cortical topology,^29^ we opted for a 20 mm diffusion kernel. All metrics were resampled to a template surface. Before downstream statistical analysis, cortical thickness, surface area, and LGI measurements were corrected for scanner-related batch effects using the Combat Tool for Harmonization of Multi-Site Imaging Data in R.^30^ Combat is a validated harmonization tool for multiscanner cross-sectional comparison of cortical thickness^31^ and surface area^30^ measurements, among others.

### Neuropsychological Evaluation

All participants underwent comprehensive neuropsychological assessment in their primary language on the same day or the day after scanning. Evaluations included standardized measures of executive function and verbal learning and self-reported questionnaires assessing mood (depression and/or anxiety). Because of discrepancies in the tests used in both centers, patients' raw scores were standardized (*z*-scores) in relation to the control group of the respective center. eAppendix 1 provides a list of used cognitive tests and self-assessment questionnaires.

### Statistical Analysis

Demographic and clinical data were analyzed with R studio (version 2023.03.0+386). One-way analysis of variance (ANOVA) was used to compare continuous clinical characteristics between subgroups. Pearson χ^2^ was used for categorical data. Post hoc tests were performed using Bonferroni-corrected pairwise comparisons. All statistic tests were performed 2-tailed.

#### Surface-Based Exploratory Analysis

Statistical analysis was performed using BrainStat for MATLAB.^32^ We first explored the main influence of age in the control group on cortical thickness, surface area, and LGI (eAppendix 2). We then applied the best-fitted age model to the corresponding vertexwise group comparison analysis of cortical thickness, surface area, and LGI, with additional adjustment for sex and, in case of surface area, total white matter volume.

With the aim to replicate the findings of the UCL group in our CDK population,^13^ surface features were compared between the patient and control groups separately in both groups (UCL and CDK) with vertexwise 2-tailed Student *t* tests (eAppendix 3). We then performed the same-group comparison in the pooled population and also compared individuals with drug-resistant JME against drug-responsive JME, as well as each patient subgroup against healthy controls. In group comparisons, a binary indicator of the group (e.g., 0 = person with drug-responsive epilepsy, 1 = person with drug-resistant epilepsy) was the predictor of interest while cortical thickness, surface area, or LGI of a specific vertex was the outcome of interest. As stated above, all surface metrics were corrected for age and sex. We calculated vertexwise effect size estimates using Cohen *d* and applied multiple comparison correction using random field theory (RFT)^33^ at a familywise error (FWE) of 0.05.

#### Neuropsychological Data

We used multivariate analysis of covariance (MANCOVA) to compare neuropsychological data between groups and included age and sex as covariates while using Wilk λ as the multivariate test statistic. For all neuropsychological tests, we compared drug-resistant individuals, drug-sensitive individuals, and healthy controls by performing univariate analysis of covariance (ANCOVA) on the z-standardized test scores while adjusting for age as a continuous variable and sex and center (CDK or UCL) as dichotomous variables. ANCOVA *p* values were corrected for multiple comparisons using false discovery rate (FDR). Pairwise *t* tests on residualized metrics (hence age-adjusted and sex-adjusted) were Bonferroni-corrected for multiple comparisons. Missing data were addressed by pairwise deletion in all analyses. We repeated the ANCOVA further adjusting for hospital anxiety and depression (Hospital Anxiety and Depression Scale—depression [HADS-A] and Hospital Anxiety and Depression Scale—depression [HADS-D]) scores.

To reduce data dimensionality, 2 principal component analyses (PCAs) were run and composite cognitive variables were obtained for (1) executive function (trail making test [TMT] B-A, digit span, lexical fluency, and semantic fluency) and (2) verbal memory (list learning and list recall). We included language fluency within the scope of executive function. The first principal component of each PCA was retained for further correlation analysis after verification that it explained a sizeable amount of the variance (>40%) and had an eigenvalue >1.

To explore the influence of disease chronicity on cognition, we conducted correlation analysis between the composite cognitive scores of executive function and verbal memory and disease duration across all individuals. Results are provided in detail in eAppendix 5.

#### Correlation Between Cognitive Domains and Brain Anatomy

To explore the relationship between brain surface architecture and executive function/verbal memory, we performed a vertexwise partial Pearson correlation analysis between each brain surface feature and the composite cognitive score of executive function and verbal memory for each participant subgroup (controls, drug-resistant participants, and drug-sensitive participants). We adjusted each correlation analysis for disease duration and total ASM score in both the drug-resistant and drug-sensitive groups and for age in the control group. Similar to our group comparison analysis, we performed multiple comparison correction with RFT at *p*_FWE_ < 0.05.

### Data Availability

The data supporting the findings of this study are available from the corresponding author on reasonable request. Study data sets are not made publicly available because of ethical and data protection restrictions.

## Results

Healthy controls and people with JME were comparable in age, sex, and center affiliation (eTable 1). In the subgroup analysis, participants with drug-resistant disease and drug-responsive disease and healthy controls differed in age at the time of scan (ANOVA *p* < 0.05). Post hoc testing revealed a significant difference between drug-responsive participants and controls (mean [SD] 34 years [10] vs 29 years [9], respectively; *p*_uncorrected_ < 0.05) and between drug-responsive and drug-resistant participants (mean [SD] 34 years [10] vs 29 years [11], respectively; *p*_uncorrected_ < 0.05). Participants with drug-resistant JME presented a higher total number of ASM trials in life than drug-sensitive participants (mean [SD] 2.17 [0.38] vs 1.69 [0.75], ANOVA *p* < 0.001), but no significantly different total ASM load (ANOVA *p* = 0.20). Center affiliation was also different between the 3 subgroups (ANOVA *p* < 0.05), with post hoc tests identifying differences between controls and drug-sensitive participants (52 [73%] vs 19 [51%] respectively; *p*_uncorrected_ < 0.05) and between participants with drug-resistant and drug-responsive JME (32 [76%] vs 19 [51%], respectively; *p*_uncorrected_ < 0.05). Both disease subgroups expressed higher rates of anxiety (median [SD] healthy controls = 3.2 [2.4] vs drug-resistant = 6.0 [3.9] vs drug-sensitive = 5.3 [3.7]; ANOVA *p* < 0.001) and depressive symptoms (median [SD] healthy controls = 2.20 [1.9] vs drug-resistant = 4.15 [2.6] vs drug-sensitive = 3.58 [3.4]; ANOVA *p* < 0.001). All subgroups were comparable for sex and prevalence of generalized tonic-clonic seizures. Demographic characteristics of study participants are provided in Table 1. Comparisons between controls and all people with JME are provided in eTable 1.

**Table 1:** Demographics, Clinical Characteristics, and Self-Assessment Questionnaires

	Controls (n = 71)	Drug-resistant (n = 42)	Drug-sensitive (n = 37)	Test statistic	*p* Value	Post hoc *p* values (uncorrected)
Demographics and clinical characteristics						
Age, y, mean (SD)						
At MRI scan	29 (9)	29 (11)	34 (10)	3.47^a^	**<0.05**	Controls/drug-resistant: >0.90 Controls/drug-sensitive: **<0.05** Drug-resistant/drug-sensitive: **<0.05**
At seizure onset		14.0 (4.3)	14.4 (3.5)	0.20	0.7	—
Female, n (%)	43 (61)	21 (50)	22 (59)	1.29^b^	0.679	—
Scanned at CDK, n (%)	52 (73%)	32 (76%)	19 (51%)	6.95^b^	**<0.05**	Controls/drug-resistant: >0.9 Controls/drug-sensitive: **<0.05** Drug-resistant/drug-sensitive: **<0.05**
Duration of epilepsy, y, mean (SD)		15 (12)	20 (11)	3.80	0.06	—
Days since last seizure, mean (SD)		184 (264)	1,812 (1,573)	42.64	**<0.001**	—
Total number of ASM trials, mean (SD)		2.17 (0.38)	1.69 (0.75)	12.89	**<0.001**	—
Total ASM load score, mean (SD)		1.35 (0.86)	1.05 (0.97)	2.05	0.20	—
Generalized tonic-clonic seizures, n (%)		39 (93)	28 (76)	3.27	0.06	—
Currently on zonisamide/topiramate, n (%)		7 (17)	4 (11)	0.18	0.528	—
Self-assessment questionnaires						
HADS-A score, mean (SD)	3.2 (2.4)	6.0 (3.9)	5.3 (3.7)	9.85^a^	**<0.001**	Controls/drug-resistant: **<0.001** Controls/drug-sensitive: **<0.05** Drug-resistant/drug-sensitive: 0.40
HADS-D score, mean (SD)	2.2 (1.90)	4.15 (2.56)	3.59 (3.39)	7.88^a^	**<0.001**	Controls/drug-resistant: **<0.001** Controls/drug-sensitive: **<0.05** Drug-resistant/drug-sensitive: 0.30

Abbreviations: ANOVA = analysis of variance; ASM = antiseizure medication; CDK = Christian Doppler University Hospital in Salzburg, Austria; HADS-A/D = Hospital Anxiety and Depression Scale—anxiety/depression; PMU = Paracelsus Medical University.

Bold indicates statistical significance.

a ANOVA test.

b Pearson χ^2^.

### Analysis of Cortical Markers

#### Individuals With JME vs Healthy Controls

**Figure 1:**
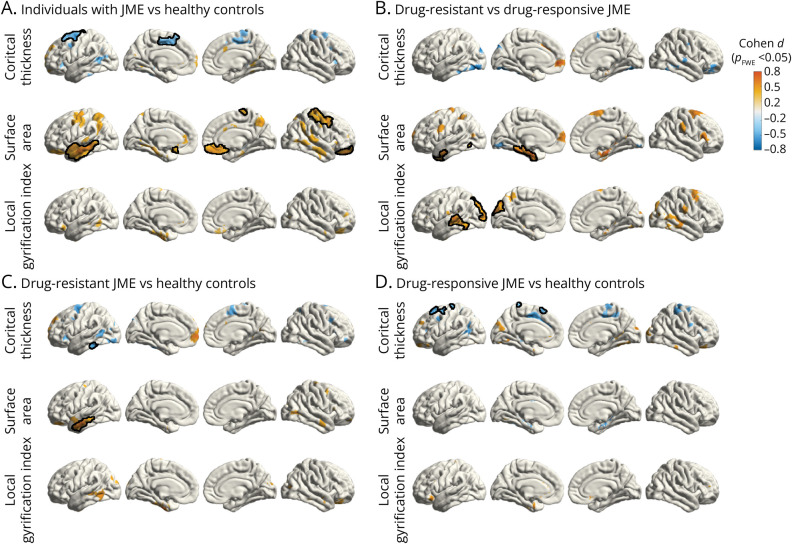
Vertexwise Group Comparisons of Surface Features Mass univariate analysis showing group comparisons of cortical thickness, surface area, and LGI (A) between individuals with JME and healthy controls (syndrome-related effects) and (B) between drug-resistant and drug-responsive individuals with JME (effects related to disease severity). Comparisons against the healthy control group are shown in (C) and (D). All surface features were corrected for age and sex while surface area was further corrected for total white matter volume. Clusters are color-coded according to the corresponding effect size estimates as reported by Cohen *d* (color bar). Clusters that survived multiple comparison correction using random field theory at *p*_FWE_ < 0.05 are outlined in black. FWE = familywise error; JME = juvenile myoclonic epilepsy; LGI = local gyrification index.

Compared with healthy controls, people with JME showed cortical thinning in the medial and superior lateral aspect of the left superior frontal gyrus. This group also presented with significantly increased surface area in the anterolateral aspect of the left temporal lobe and in the right orbitofrontal cortex and precentral and postcentral gyrus (Figure 1A). No changes in LGI were observed between individuals and healthy controls.

#### Individuals With Drug-Resistant vs Drug-Responsive JME

Individuals with drug-resistant JME showed increased white matter surface area in multiple loci of the medial and lateral aspects of the left prefrontal and parietal cortices and the medial temporal lobes. Clusters in the medial left temporal lobe and left temporal pole survived familywise correction (Figure 1B). LGI was increased in the lateral aspect of the temporal lobes and occipital cortex bilaterally in drug-resistant individuals, with clusters on the left side surviving familywise correction (Figure 1B). We observed no significant abnormalities in cortical thickness between groups.

#### Individuals With Drug-Resistant and Drug-Sensitive JME vs Healthy Controls

Individuals with drug-resistant JME exhibited diffuse left-weighted cortical thinning, surviving familywise multiple comparison correction in the left inferior temporal gyrus (Figure 1C), compared with healthy controls. In addition, surface area was increased along the lateral aspect of the left temporal lobe. When contrasting drug-responsive participants with healthy controls, we observed significant cortical thinning in localized areas within the left precentral gyrus and superior frontal gyrus, particularly emphasized on the left hemisphere. No differences in surface area or LGI were observed between drug-responsive individuals and healthy controls (Figure 1D).

Repeat patient subgroup analysis further covarying for total ASM load and handedness separately did not alter the results.

### Cognitive Performance Across Groups

#### Neuropsychological Test *z*-Scores

Complete neuropsychological assessment was available for a total of 69 participants with JME (36 of whom had drug-resistant disease and 33 with drug-sensitive disease) and 59 healthy controls. MANCOVA covarying for age, sex, and center yielded a significant effect of group on cognitive performance (Wilk λ = 0.65, *F* = 3.90, *p* < 0.001). Subsequent ANCOVAs adjusted for sex, age, and center demonstrated significant group differences in TMT B-A (*p*_FDR_ < 0.01), TMT A (*p*_FDR_ < 0.001), digit span backward (*p*_FDR_ < 0.01), verbal learning (*p*_FDR_ < 0.01), letter fluency (*p*_FDR_ = 0.001), and word fluency (*p*_FDR_ = 0.001). Repeat analysis further correcting for either anxiety or depression self-assessment scores did not alter the significance of results. Post hoc tests showed worse performance of drug-resistant participants against controls in all cognitive tests except for verbal recall (for letter fluency and TMT A, *p*_Bonferroni_ < 0.001; for digit span backward, word fluency, and verbal learning, *p*_Bonferroni_ < 0.01; for TMT B-A, *p*_Bonferroni_ = 0.05). This group also performed worse than drug-sensitive participants in the TMT A (*p*_Bonferroni_ = 0.001), digit span backward (*p*_Bonferroni_ < 0.05), letter fluency (*p*_Bonferroni_ < 0.05), and word fluency (*p*_Bonferroni_ < 0.01). Detailed statistics regarding neuropsychological test outcomes are provided in Table 2.

**Table 2:** Performance in Neuropsychological Tests Across Groups (*z*-Scores)

	Drug-resistant (n = 36)	Drug-responsive (n = 33)	*F* Statistic	*p*_FDR_ Value	Post hoc *p* values^a^ (Bonferroni-corrected)
Executive function					
TMT B-A	1.00 (0.32)	0.66 (0.25)	6.77	**<0.01**	Controls/drug-resistant: **<0.05** Controls/drug-responsive: >0.90 Drug-resistant/drug-responsive: 0.20
Digit span backward	−0.46 (0.15)	0.30 (0.15)	6.39	**<0.01**	Controls/drug-resistant: **<0.01** Controls/drug-responsive: 0.90 Drug-resistant/drug-responsive: **<0.05**
Letter fluency	−0.91 (0.19)	−0.30 (0.18)	8.88	**0.001**	Controls/drug-resistant: **<0.001** Controls/drug-responsive: 0.60 Drug-resistant/drug-responsive: **<0.05**
Word fluency	−0.96 (0.22)	0.03 (0.27)	7.87	**0.001**	Controls/drug-resistant: **<0.01** Controls/drug-responsive: >0.90 Drug-resistant/drug-responsive: **<0.01**
Psychomotor speed					
TMT A	1.09 (0.28)	0.12 (0.18)	10.61	**<0.001**	Controls/drug-resistant: **<0.001** Controls/drug-responsive: 0.90 Drug-resistant/drug-responsive: **0.001**
Verbal memory					
Verbal learning	−0.81 (0.20)	−0.43 (0.19)	6.99	**<0.01**	Controls/drug-resistant: **<0.01** Controls/drug-responsive: 0.70 Drug-resistant/drug-responsive: 0.30
Verbal recall	−0.03 (0.17)	−0.09 (0.15)	0.19	0.8	

Abbreviations: FDR = false discovery rate; MANCOVA = multivariate analysis of covariance; TMT = trail making test.

Neuropsychological test scores are presented as mean *z*-scores (SD) after standardization in relation to the respective control group (in total n = 59).

Bold indicates statistical significance.

a Post hoc *p* values are calculated from residualized metrics after covarying for age, sex, and center.

#### Neuropsychological Domains

**Figure 2:**
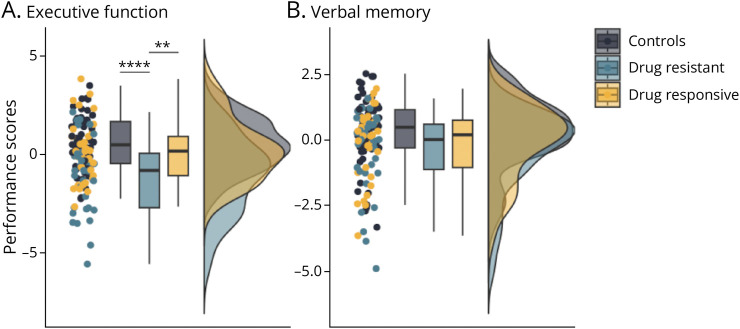
Performance in Neuropsychological Domains Across Groups Raincloud plots show the distribution of composite cognitive construct scores representing (A) executive function and (B) verbal memory performance across healthy controls and individuals with drug-resistant and drug-responsive JME. In A and B, asterisks refer to *p* values for Bonferroni-corrected, age-adjusted, and sex-adjusted post hoc *t* tests calculated on the residuals from the analysis of covariance. Statistical details are provided in Table 3. **p < 0.01, ***p < 0.001, ****p < 0.0001.

The first PCs of executive function and verbal memory had eigenvalues of 3.16/2.01 and explained 56.02%/89.62% of the total variance, respectively. Loadings of both PCAs are provided in eTable 2. MANCOVA revealed a significant effect of group on cognitive performance based on the cognitive composite scores of executive function and verbal memory (Wilk λ = 0.82, *F* = 6.44, *p* < 0.01) covarying for age, sex, and center. ANCOVA with age, sex, and center as covariates showed significant group differences in both executive function and verbal memory (*p*_FDR_ < 0.001 and *p*_FDR_ < 0.05, respectively). Post hoc *t* tests on residualized metrics after adjustment for age, sex, and center showed worse executive function performance in drug-resistant individuals against controls (*p*_Bonferroni_ < 0.001) and drug-responsive individuals (*p*_Bonferroni_ < 0.01) while no difference was found between drug-sensitive participants and controls. Concerning performance in verbal memory, no intergroup significant differences were found after correction for multiple comparisons. Repeated ANCOVA covarying further for either HADS-A or HADS-D scores did not alter the significance of results. The effects of group on domain-specific neuropsychological performance are presented in Figure 2 and provided in more detail in Table 3.

**Table 3:** Performance in Neuropsychological Domains Across Groups (Composite Scores)

	Controls (n = 59)	Drug-resistant (n = 36)	Drug-responsive (n = 33)	*F* Statistic	*p*_FDR_ Value	Post hoc *p* values^a^ (Bonferroni-corrected)
Executive function	0.61 (0.17)	−1.13 (0.35)	0.16 (0.28)	13.11	**<0.001**	Controls/drug-resistant: **<0.001** Controls/drug-responsive: 0.90 Drug-resistant/drug-responsive: **<0.01**
Verbal memory	0.33 (0.17)	−0.44 (0.27)	−0.01 (0.25)	4.51	**<0.05**	Controls/drug-resistant: 0.30 Controls/drug-responsive: 0.90 Drug-resistant/drug-responsive: 0.20

Abbreviations: FDR = false discovery rate; MANCOVA = multivariate analysis of covariance.

Principal component analysis–derived composite cognitive mean scores (SD) respective to executive function and verbal memory are presented.

Bold indicates statistical significance.

a Post hoc *p* values are calculated from residualized metrics after covarying for age, sex, and center.

### Correlation Between Neuropsychological Domains and Cortical Surface Features

Complete neuropsychological assessment and structural brain imaging was available for 35 participants with drug-resistant JME, 31 with drug-sensitive disease, and 58 healthy controls.

#### Executive Function

**Figure 3:**
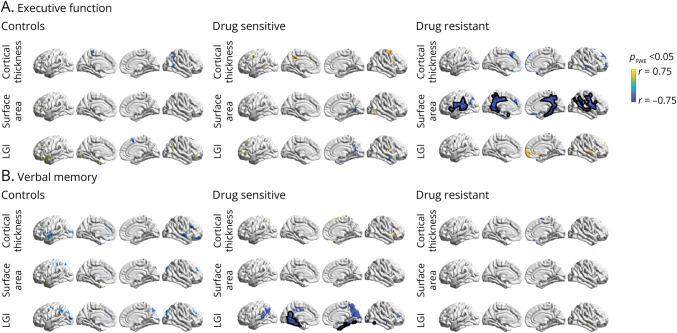
Correlation Between Neuropsychological Domains and Cortical Surface Features Vertexwise partial Pearson correlation plots between the composite cognitive constructs for executive function (A), verbal memory (B), and the cortical surface features (cortical thickness, surface area, and LGI). Partial correlation analysis used age and sex as covariates for healthy controls and disease duration, sex, and total ASM load for the drug-sensitive and drug-resistant groups. Significant clusters that survived multiple comparison corrections using random field theory at *p*_FWE_ < 0.05 are outlined in black. Clusters are color-coded according to the partial Pearson correlation coefficient (*r*) (color bar). ASM = antiseizure medication; FWE = familywise error; JME = juvenile myoclonic epilepsy; LGI = local gyrification index.

In the drug-resistant group, we observed a correlation of worse performance in executive function with greater surface area within the posterior cingulum and precuneus bilaterally, the medial temporal lobe (right more than left), and the lateral aspects of the temporal and frontal lobes in a diffuse symmetrical distribution (all *p*_FWE_ < 0.05) after controlling for disease duration and total ASM load (Figure 3A). In the drug-responsive and control groups, no vertices survived familywise correction for the same partial correlation analysis.

#### Verbal Memory

When investigating the association between verbal memory performance and surface metrics, partial correlation analysis highlighted worse verbal memory with greater LGI of the medial temporal lobes in the drug-sensitive group, after covarying for disease chronicity and ASM load (*p*_FWE_ < 0.05) (Figure 3B). No significant correlations were detected in the control and drug-resistant group.

## Discussion

In this cross-sectional MRI study, we observed increased white matter surface area and folding complexity with left-sided predominance in participants with drug-resistant JME compared with those with drug-sensitive JME. Participants with drug-resistant JME also showed more pronounced deficits on executive function tests, the extent of which correlated with the severity of identified structural changes.

Cortical folding, or gyrification, is a dynamic developmental process in which the cerebral cortex expands in volume and space, accompanied by complex tissue folding.^34,35^ Closely linked to the gyrification process is the white matter surface area, a well-established developmental marker that reflects the tangential expansion of cortical columns within a region, contributing to the extent of cortical folding.^36^ Abnormalities in cortical expansion and, consequently, gyrification, have repercussions on the resulting system-level connectivity^37^ and can manifest clinically in neuropsychiatric and neurologic disorders. Notably, studies in children with autism spectrum disorder^38^ and in primates with prenatal brain injury^39^ showed an association between locally altered gyrification patterns and underlying decreased structural and functional connectivity.

Previous neuroimaging studies have not delivered solid evidence of a relationship between structural abnormalities and disease severity in IGE syndromes.^40-42^ In our pooled population, we highlighted disease-related abnormalities in cortical surface area and folding complexity by comparing healthy controls and people with JME. In this study, white matter surface area was increased in the patient group bilaterally and cortical thinning was limited to the premotor and medial prefrontal cortex on the left side. Although these patterns were mostly driven by the UCL population, we found similar findings in the CDK group by observing a tendency for diffusely expanded surface area and thinned cortex associated with JME. We then investigated the structural phenotype associated with a more severe clinical course by comparing individuals with drug resistance with those with controlled disease. Clinical severity was associated with diffusely increased white matter surface and LGI in frontal, temporal, and parietal lobes. These changes survived familywise correction in the left medial temporal (surface area) and left lateral temporal lobe and occipital cortex (LGI). No discernible alterations in surface area and LGI were observed after comparing drug-responsive participants and healthy controls, suggesting that the drug-resistant group is mainly driving these abnormalities. As previously reported, not only white matter surface area and gyrification are genetically determined cortical features, but also the former has been established in part of our study cohort as an endophenotype in JME after comparison with unaffected siblings.^13^ We thus hypothesize that the abnormalities of cortical architecture observed in our refractory disease group reflect a postmigrational developmental signature of drug-resistant JME, which impairs the network circuitry and ultimately contributes to the observed clinical phenotype with drug resistance and cognitive impairment. This likely represents a subtle structural phenotype because only minimal changes were detected when contrasting drug-resistant participants with healthy controls. Our findings thus support a neurodevelopmental network basis as the cause of drug resistance in IGE syndromes, opposing altered ASM kinetics as proposed earlier.^9,10^ The overlap between surface area and cortical folding abnormalities on the temporal lobes aligns with previous findings on left-weighted functional and structural changes in JME.^19,20^ Consistent with these studies, we found a higher weight of left hemisphere abnormalities after correction for handedness here also. We hypothesize that this may relate to the longer maturation time of the left hemisphere, rendering it more susceptible to developmental stressors.^43^ This, in turn, may be underlain by asymmetric gene expression profiles regulating asymmetric development of human brains.^44^

In contrast to sulcal-gyral markers, evidence for altered cortical thickness patterns in IGEs is more conflicting. Recent large-scale studies showed a predilection of the precentral cortices for atrophy, not only in individuals with JME^13,45^ but also across all epilepsy types.^15^ In this study, we reproduced previous findings from large-scale studies in IGEs by showing gray matter loss in the left premotor cortex. Of interest, cortical thinning in these areas was not associated with more severe disease but was seemingly more pronounced in drug-responsive participants. These findings thus challenge the concept of cortical thinning as a marker of disease severity within IGEs and further suggest a divergent developmental trajectory between drug-resistant and drug-responsive JME.

Changes in network connectivity have been found in individuals with IGEs with uncontrolled seizures and are likely to drive a more severe clinical phenotype including cognitive impairment and psychiatric comorbidity.^46,47^ We explored the neurocognitive profile of our cohort by adopting a dimensionality reduction approach and focusing on neurocognitive domains, in line with recent literature.^25^ A more pronounced impairment of both executive function and verbal memory was associated with longer disease duration independently of ASM load or disease onset, thereby revealing a relationship between multidomain cognitive performance and disease chronicity. Nevertheless, after adjusting for the effects of age, drug-resistant disease was still associated with a higher difficulty in executive function tasks when compared with both drug-responsive individuals and controls. These findings show consistency with previous studies as they suggest executive dysfunction as a core characteristic of the neuropsychological phenotype of people with drug-resistant JME.^6,48^

Our whole-brain partial correlation analysis revealed a set of cortical areas comprising the posterior cingulum, the precuneus, the medial temporal lobe, and peri-Sylvian regions, where the extent of surface area abnormalities showed an association with worse executive function. The spatial distribution of this relationship largely overlapped with cortical regions that demonstrated increased surface area in drug-resistant individuals when compared with drug responders. In light of these findings, we can infer not only that white matter surface area is increased in the medial temporal and prefrontal areas as part of a structural neurodevelopmental phenotype specific to drug-resistant JME but also that the extent of such phenotypic structural changes correlates with executive function performance; that is, the more abnormal the structural phenotype (as represented by surface area increase), the higher the impairment in executive function. This relationship, however, only seems to hold true for drug-resistant disease. Of interest, drug-responsive disease showed a divergent cognitive developmental profile, where increasing folding complexity in the medial temporal lobes bilaterally was associated with deficits in verbal memory, but not in executive function, despite preserved verbal memory performance in drug-responsive disease. Indeed, not only is it evident that cortical architectural changes reflect widespread network disorder by extending beyond the prefrontal lobe to the medial temporal and parietal areas, among others, but also they do so in seemingly different patterns between drug-resistant and drug-responsive JME. This may reflect distinct network reorganization because of divergent neurodevelopmental trajectories between both disease subgroups, further emphasizing the structural heterogeneity of JME and the possibility that we are looking at different diseases. We controlled for a potential influence of ASM-related effects on cortical morphology by covarying these analyses to the total ASM load.

Our study has limitations. Given the cross-sectional design, we cannot prove whether the observed structural abnormalities are causative changes, consequences of seizure activity, due to prolonged drug treatment, or multifactorial. Epilepsies are associated with dynamic structural changes and, therefore, warrant longitudinal long-term studies to capture the spatiotemporal evolution of cortical architecture in IGEs and, as emphasized by our results, also its asymmetric patterns. In line with this notion, normal aging is believed to offset the observed developmental abnormalities in JME at some point in life, translating into the clinical benign course of the disease (in layperson terms, patients “grow out of their disease”). This is supported by clinical evidence demonstrating improved seizure control typically starting around the fourth decade of life onward, coinciding with presumed completion of brain maturation.^49^ Given that our drug-responsive participants are notably older than the drug-resistant counterparts and despite correction for age as a surrogate marker of brain maturation, it remains plausible that some participants in the drug-responsive group may have been classified as drug resistant had they been scanned earlier in their disease course. Another limitation arises from the complex interplay of antiseizure medication and recurrent seizure activity on cognition, which adds increased difficulty in identifying a distinct neuropsychological profile in drug-resistant JME. Furthermore, pseudo-drug resistance can occur in up to 20% of individuals with JME and is challenging to account for.^50^ Owing to lacking information regarding patient adherence to medication, some individuals may have been misclassified as drug resistant in our study.

In conclusion, we identified distinct and divergent cognitive developmental phenotypes for drug-resistant and drug-responsive JME, supporting a neurodevelopmental basis for disease severity in IGE syndromes. We provide new insights into the pathophysiology of IGEs and deliver a developmental framework on which future genetic, imaging, and clinical studies can build on. Should our findings be translated as an imaging biomarker of drug-resistant IGE, the early identification of individuals at risk of developing drug resistance through MRI screening and phenotypical prediction models could improve patient care by facilitating prompt and tailored pharmacologic and cognitive interventions, for example, such as justifying early treatment with sodium valproate.

## Appendix Authors

**Table:**

Name	Location	Contribution
Bernardo Crespo Pimentel, MD	Department of Neurology, Neurointensive Care and Neurorehabilitation, Christian Doppler University Hospital, Paracelsus Medical University, Centre for Neuroscience Salzburg, Member of the European Reference Network, EpiCARE, Austria; Department of Clinical & Experimental Epilepsy, UCL Queen Square Institute of Neurology, London; Chalfont Centre for Epilepsy, Chalfont St. Peter, United Kingdom; Neuroscience Institute, Christian-Doppler University Hospital, Centre for Cognitive Neuroscience, Salzburg, Austria	Drafting/revision of the manuscript for content, including medical writing for content; study concept or design; analysis or interpretation of data
Giorgi Kuchukhidze, MD, PhD	Department of Neurology, Neurointensive Care and Neurorehabilitation, Christian Doppler University Hospital, Paracelsus Medical University, Centre for Neuroscience Salzburg, Member of the European Reference Network, EpiCARE; Neuroscience Institute, Christian-Doppler University Hospital, Centre for Cognitive Neuroscience, Salzburg, Austria	Major role in the acquisition of data; study concept or design
Fenglai Xiao, MD, PhD	Department of Clinical & Experimental Epilepsy, UCL Queen Square Institute of Neurology, London; Chalfont Centre for Epilepsy, Chalfont St. Peter, United Kingdom	Major role in the acquisition of data; study concept or design
Lorenzo Caciagli, MD, PhD	Department of Clinical & Experimental Epilepsy, UCL Queen Square Institute of Neurology, London; Chalfont Centre for Epilepsy, Chalfont St. Peter, United Kingdom; Department of Neurology, Inselspital, Sleep-Wake-Epilepsy-Center, Bern University Hospital, University of Bern, Switzerland	Major role in the acquisition of data; study concept or design
Julia Hoefler, MD	Department of Neurology, Neurointensive Care and Neurorehabilitation, Christian Doppler University Hospital, Paracelsus Medical University, Centre for Neuroscience Salzburg, Member of the European Reference Network, EpiCARE; Neuroscience Institute, Christian-Doppler University Hospital, Centre for Cognitive Neuroscience, Salzburg, Austria	Major role in the acquisition of data
Lucas Rainer, MSc, PhD	Department of Neurology, Neurointensive Care and Neurorehabilitation, Christian Doppler University Hospital, Paracelsus Medical University, Centre for Neuroscience Salzburg, Member of the European Reference Network, EpiCARE; Department of Child and Adolescent Psychiatry, Christian Doppler University Hospital, Paracelsus Medical University, Salzburg, Austria	Major role in the acquisition of data
Martin Kronbichler, BSc, PhD	Neuroscience Institute, Christian-Doppler University Hospital, Centre for Cognitive Neuroscience, Salzburg; Department of Psychology, University of Salzburg, Austria	Major role in the acquisition of data
Christian Vollmar, MD, PhD	Department of Neurology, Epilepsy Center, University Hospital of the Ludwig-Maximilians-University of Munich, Germany	Major role in the acquisition of data
John S. Duncan, FRCP, FMedSci	Department of Clinical & Experimental Epilepsy, UCL Queen Square Institute of Neurology, London; Chalfont Centre for Epilepsy, Chalfont St. Peter, United Kingdom	Major role in the acquisition of data
Eugen Trinka, MD, MSc, FRCP	Department of Neurology, Neurointensive Care and Neurorehabilitation, Christian Doppler University Hospital, Paracelsus Medical University, Centre for Neuroscience Salzburg, Member of the European Reference Network, EpiCARE; Neuroscience Institute, Christian-Doppler University Hospital, Centre for Cognitive Neuroscience, Salzburg; Department of Public Health, Health Services Research and Health Technology Assessment, UMIT-University of Health Sciences, Medical Informatics and Technology, Hall in Tirol; Karl Landsteiner Institute for Neurorehabilitation and Space Neurology, Salzburg, Austria	Major role in the acquisition of data; study concept or design
Matthias J. Koepp, MD, PhD, FRCP	Department of Clinical & Experimental Epilepsy, UCL Queen Square Institute of Neurology, London; Chalfont Centre for Epilepsy, Chalfont St. Peter, United Kingdom	Drafting/revision of the manuscript for content, including medical writing for content; major role in the acquisition of data; study concept or design; analysis or interpretation of data
Britta Wandschneider, MD, PhD	Department of Clinical & Experimental Epilepsy, UCL Queen Square Institute of Neurology, London; Chalfont Centre for Epilepsy, Chalfont St. Peter, United Kingdom	Drafting/revision of the manuscript for content, including medical writing for content; major role in the acquisition of data; study concept or design; analysis or interpretation of data

## Glossary

3D: 3-dimensional
ANCOVA: analysis of covariance
ANOVA: analysis of variance
ASM: antiseizure medication
CDK: Christian Doppler University Hospital in Salzburg, Austria
FDR: false discovery rate
FWE: familywise error
HADS-A/D: Hospital Anxiety and Depression Scale—anxiety/depression
IGE: idiopathic generalized epilepsy
JME: juvenile myoclonic epilepsy
LGI: local gyrification index
MANCOVA: multivariate analysis of covariance
PCA: principal component analysis
RFT: random field theory
TMT: trail making test
UCL: University College London Hospitals, London, United Kingdom
